# Understanding Community Health Care Through Problem-Based Learning With Real-Patient Videos: Single-Arm Pre-Post Mixed Methods Study

**DOI:** 10.2196/68743

**Published:** 2025-01-31

**Authors:** Kiyoshi Shikino, Kazuyo Yamauchi, Nobuyuki Araki, Ikuo Shimizu, Hajime Kasai, Tomoko Tsukamoto, Hiroshi Tajima, Yu Li, Misaki Onodera, Shoichi Ito

**Affiliations:** 1 Chiba University Graduate School of Medicine Community-Oriented Medical Education Chiba Japan; 2 Health Professional Development Center Chiba University Hospital Chiba University Chiba Japan; 3 Department of General Medicine Chiba University Hospital Chiba University Chiba Japan; 4 Department of Medical Education Chiba University Graduate School of Medicine Chiba University Chiba Japan

**Keywords:** community health care, community-oriented medical education, mixed method, problem-based learning, real-patient video

## Abstract

**Background:**

Japan faces a health care delivery challenge due to physician maldistribution, with insufficient physicians practicing in rural areas. This issue impacts health care access in remote areas and affects patient outcomes. Educational interventions targeting students’ career decision-making can potentially address this problem by promoting interest in rural medicine. We hypothesized that community-based problem-based learning (PBL) using real-patient videos could foster students’ understanding of community health care and encourage positive attitudes toward rural health care.

**Objective:**

This study investigated the impact of community-based PBL on medical students’ understanding and engagement with rural health care, focusing on their knowledge, skills, and career orientation.

**Methods:**

Participants were 113 fourth-year medical students from Chiba University, engaged in a transition course between preclinical and clinical clerkships from October 24 to November 2, 2023. The students were randomly divided into 16 groups (7-8 participants per group). Each group participated in two 3-hour PBL sessions per week over 2 consecutive weeks. Quantitative data were collected using pre- and postintervention questionnaires, comprehension tests, and tutor-assessed rubrics. Self-assessment questionnaires evaluated the students’ interest in community health care and their ability to envision community health care settings before and after the intervention. Qualitative data from the students’ semistructured interviews after the PBL sessions assessed the influence of PBL experience on clinical clerkship in community hospitals. Statistical analysis included median (IQR), effect sizes, and P values for quantitative outcomes. Thematic analysis was used for qualitative data.

**Results:**

Of the 113 participants, 71 (62.8%) were male and 42 (37.2%) female. The total comprehension test scores improved significantly (pretest: median 4.0, IQR 2.5-5.0; posttest: median 5, IQR 4-5; *P*<.001; effect size r=0.528). Rubric-based assessments showed increased knowledge application (pretest: median 8, IQR 7-9; posttest: median 8, IQR 8-8; *P*<.001; r=0.494) and self-directed learning (pretest: median 8, IQR 7-9; posttest: median 8, IQR 8-8; *P*<.001; r=0.553). Self-assessment questionnaires revealed significant improvements in the students’ interest in community health care (median 3, IQR 3-4 to median 4, IQR 3-4; *P*<.001) and their ability to envision community health care settings (median 3, IQR 3-4 to median 4, IQR 3-4; *P*<.001). Thematic analysis revealed key themes, such as “empathy in patient care,” “challenges in home health care,” and “professional identity formation.”

**Conclusions:**

Community-based PBL with real-patient videos effectively enhances medical students’ understanding of rural health care settings, clinician roles, and the social needs of rural patients. This approach holds potential as an educational strategy to address physician maldistribution. Although this study suggests potential for fostering positive attitudes toward rural health care, further research is needed to assess its long-term impact on students’ career trajectories.

## Introduction

Japan faces a significant health care delivery challenge owing to uneven physician distribution, notably affecting rural areas and community hospitals [1,2]. This maldistribution exacerbates community hospitals’ challenges [3-5]. This issue is not confined to Japan; it impacts countries worldwide [6-11]. In 2019, the Ministry of Health, Labour and Welfare introduced the physician uneven distribution index as part of an intervention policy addressing prefectural geographical disparities in physician distribution [1,12-14]; it assesses the extent of physician maldistribution by evaluating prefectural medical supply and demand.

To combat the physician maldistribution, community hospital training has been integrated into second-year resident physicians’ compulsory curriculum [15-17], highlighting the necessity of preparing future physicians with the competencies required to effectively meet rural communities’ health care needs. Moreover, introducing community medicine principles early in medical education is an acknowledged need [18].

Japanese medical schools have begun to proactively adopt problem-based learning (PBL) as a foundational step before clinical rotations [19]. PBL emphasizes real-life medical scenarios, cultivating students’ clinical reasoning and decision-making skills [20]. PBL prepares students for clinical rotations with an enriched understanding of community health care’s challenges and prospects [21]. This approach bolsters medical students’ clinical training and supports the alleviation of physician maldistribution by promoting community or rural medicine careers.

However, although PBL has been implemented in medical education settings, its integration with community-oriented medicine in addressing physician maldistribution remains underexplored. Furthermore, despite the global relevance of physician maldistribution, studies focusing on innovative educational interventions targeting this issue are limited [22-24].

We hypothesized that incorporating real-patient videos into community-focused PBL would significantly improve students’ capacity to make well-informed career choices and identify with positive role models. This study addressed the aforementioned research gap by examining this approach’s effectiveness within Japanese medical education.

## Methods

### Study Design

This study used an explanatory sequential mixed methods design following a pragmatic approach [25-27], capitalizing on quantitative and qualitative designs’ strengths while minimizing their shortcomings. Furthermore, it allowed researchers to better understand experimental results while incorporating participants’ perspectives. The National Institutes of Health advises a mixed methods approach “to improve the quality and scientific power of data” and to better address the complex issues facing health sciences today, including health professional education [28,29]. In the qualitative analysis, medical students’ reflection papers were text-mined to analyze the word frequency in community-oriented PBL. Next, we conducted individual interviews with medical students during their clinical clerkship.

### Participants and Trial Design

This study was conducted as part of the undergraduate medical curriculum at Chiba University, Japan. Community-oriented PBL was conducted from October 24 to November 2, 2023, as part of a preclerkship/clerkship transition course [30], a 5-week preparatory education period before clinical clerkship. Additionally, PBL is integrated into the Chiba University medical school’s curriculum in years 1-4, and all students experience it. Participants were 113 fourth-year medical students who had attended lectures and received simulation training in basic and clinical medicine. To minimize potential biases and ensure an even distribution of student characteristics, participants were randomly divided into 16 groups of 7-8 each using the random number generation function in Microsoft Excel. The 16 groups received community-oriented PBL of a patient case using a community-based integrated care system with real-patient videos.

**Figure 1:**
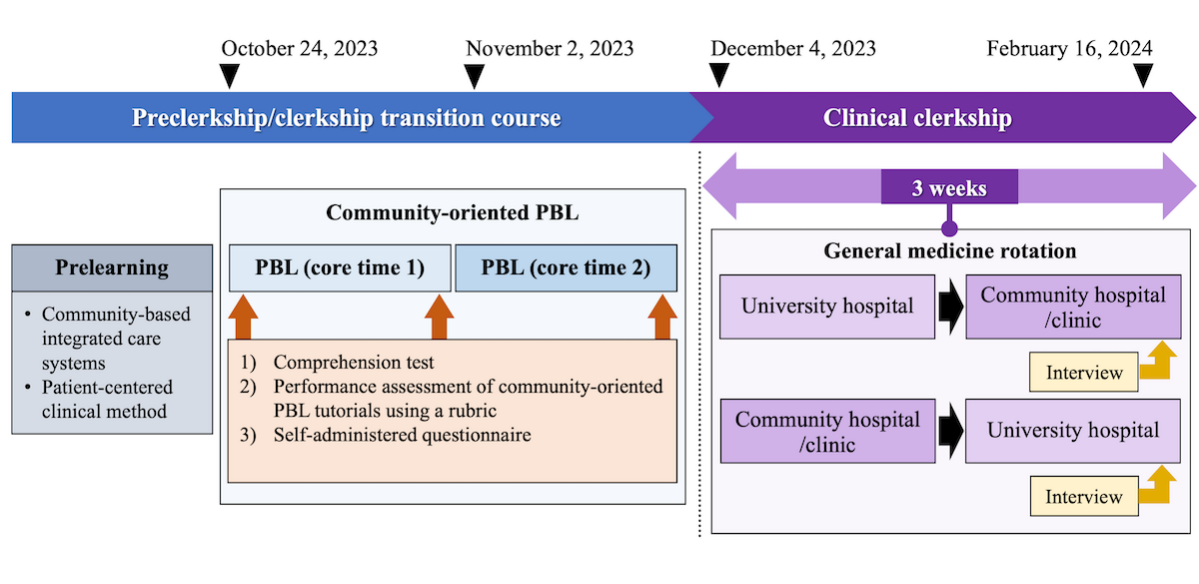
Timeline from PBL in preclerkship/clerkship transition course to general medicine rotation in clinical clerkship. PBL: problem-based learning.

Quantitative data were gathered using the comprehension test, a tutor assessment with rubrics during the core time, and pre- and postintervention questionnaires to assess community health care perceptions (Figure 1). Additionally, a qualitative evaluation assessed community health care perceptions using a free-text reflection paper after PBL and follow-up interviews during clinical clerkships in community hospitals or clinics. The study used a mixed methods, sequential explanatory design to integrate the results [25,27,31].

### Community-Oriented PBL Educational Intervention

Real-patient videos were meticulously prepared to enhance the learning experience authenticity, simulating real-life scenarios in community health care settings (Multimedia Appendix 1). Prepared in collaboration with medical education experts, community health care professionals, and audiovisual production specialists, these videos aimed to accurately depict home health care characteristics, including medical interviews, physical examinations, and in-home patient interactions. Real patients participated in the production process under strict ethical guidelines to ensure authenticity and respect for patient privacy, emphasizing community health care’s unique challenges and dynamics.

Each real-patient video was carefully scripted and filmed to represent common community health care situations, encompassing multiple scenes depicting different patient care stages and interactions, ranging from initial patient assessments in a community hospital setting to follow-up in-home visits. The video durations ranged from 3 to 5 minutes. Along with the videos, patient information sheets and tasks were presented to the students (Multimedia Appendix 2). One of the patient’s primary conditions was underlying diabetes, and they presented with severe lower leg edema. The case involved transitioning from acute care to a chronic care hospital, followed by the introduction of home visit medical services. Patient consent was obtained for video use, and students were instructed to adhere to confidentiality guidelines.

During the community-oriented PBL sessions, students collectively viewed the real-patient videos in designated classrooms equipped with audiovisual facilities, allowing for simultaneous viewing on shared screens. Before watching the videos, students were divided into small groups and assigned a tutor to facilitate discussions and learning activities. The tutors observed whether the students could achieve the learning objectives and facilitated discussions. The tutors, randomly selected faculty members in medical education and community-oriented medical education, were given standardized instructions and materials before the sessions to ensure consistency and effectiveness [32].

Community-oriented PBL sessions were divided into 2 sessions per case, each lasting approximately 3 hours. In the first session (core time 1), students were presented with the patient’s history and physical examination findings. In the second session (core time 2), the investigation findings and treatment plans were discussed. The same case scenario was used for all students.

### Quantitative Measures

#### Comprehension Test

The comprehension test assessed the minimum essential knowledge required for problem solving in PBL, focusing on holistic medicine, patient-centered care, and the International Classification of Functioning, Disability, and Health (ICF). It comprised 5 multiple-choice questions (Q1-Q5, Multimedia Appendix 3). The test was administered as a pretest at the beginning of core time 1 and a posttest after core time 2, allowing for a comparison of test scores.

The comprehension test items were developed specifically for this study and have not been used in other contexts. They were based on the core learning objectives of the PBL sessions, which included understanding the structure of community health care systems, application of the ICF, and principles of patient-centered care. To ensure clarity and alignment with learning objectives, the questions underwent cognitive debriefing by faculty members in medical education. This process involved reviewing each question for relevance, accuracy, and comprehensibility, with feedback incorporated into the final version to enhance content validity.

#### Rubric-Based Performance Assessment

In addition to the comprehension test, each student’s performance during the PBL sessions was assessed using a rubric. The rubric was determined based on previous studies after the authors discussed the validity of the criteria [33,34]. It evaluated 5 key dimensions: knowledge application, comprehensive care process, self-regulated learning, learning motivation, and communication skills with peers. Self-regulated learning refers to the ability of students to plan, monitor, and reflect on their learning process, fostering autonomy and adaptability in problem-solving contexts [35]. Each of the 5 dimensions was quantitatively evaluated on a 10-point scale (Multimedia Appendix 4). Performance assessments were conducted before and after the educational intervention to measure changes in the competencies.

#### Self-Administered Questionnaire

Students completed questionnaires before and after community-oriented PBL (Multimedia Appendix 5). They were assigned identification numbers to preserve their anonymity. Data were collected using a self-administered 5-point Likert-scale questionnaire ranging from 1 (strongly disagree) to 5 (strongly agree). The criteria were informed by previous studies and refined by the authors through discussions in focus groups [36,37]. After community-oriented PBL, 2 items (“I am interested in community health care” and “I can envision a community health care setting”) were surveyed. The items assessed the students’ interest in community health care and their ability to visualize a community health care setting.

### Sample Size

This study also served as an educational program for fourth-year medical students in a basic clinical clerkship course. Altogether, 113 medical students from 12 groups were recruited. For quantitative data, the sample size required a 2-tailed *t* test of the difference between the pre- and post-PBL means, assuming a significance level of .05, a power of 0.8, and an effect size of 0.5. When the Mann-Whitney *U* test was conducted with those values, the required sample size was 54 in each group, totaling 108.

### Data Analysis

All statistical analyses of quantitative data were conducted using SPSS Statistics for Microsoft Windows version 29.0 (IBM Corp), with a significance level under 5% for each analysis. The comprehension test results, including total scores and individual question responses (Q1-Q5), were analyzed using the Wilcoxon signed rank test for paired total scores. Additionally, the McNemar test was used to compare pre- and post-PBL correct response rates for individual questions. For rubric-based performance assessment, the Wilcoxon signed rank test was used to compare scores from core time 1 and core time 2 for total scores and individual rubric items. Effect sizes were calculated for all analyses: r values were derived from z scores for the Wilcoxon signed rank test, and the Cohen w value was calculated for the McNemar test.

### Qualitative Measures

#### Follow-Up Interviews During Clinical Clerkships

Semistructured interviews (average duration: 20 minutes) with individual medical students were conducted by authors KS, KY, and NA. All sessions were recorded and transcribed verbatim, and interviews were conducted iteratively. An interview guide containing open-ended questions was constructed deductively based on the research question and thematic analysis findings. This guide was modified after the first 9 interviews to address emerging and previously unexplored themes in subsequent interviews. The interview participants received no gifts for participating.

Interview transcripts were analyzed using a template analysis approach [38,39]. An inductive code template was defined based on the research questions, thematic analysis findings, and interview guide. The initial template was developed through independent coding (performed by authors KS and IS) of the first 9 interviews. The template was further developed by coding the subsequent interviews. Regarding version 2 of the template, after coding 3 interviews, KS, NA, and IS agreed that the template adequately covered all texts. KS and IS individually coded the remaining transcripts using the template. At this stage, authors KY and SI discussed all further changes or additions to the template until they reached a consensus. After coding all 12 interviews, no additional changes were made to the templates. The final code template was further confirmed by analyzing the remaining 12 transcripts, which can be interpreted as a sign that code saturation was reached [40].

A qualitative evaluation was conducted to assess the acquisition of higher-order intellectual skills in which an interview was conducted after PBL and clinical clerkship. In the clinical clerkship interview, community-oriented PBL’s effectiveness in improving clinical performance in home visit care was investigated. The interviewers (KS, KY, and NA) discussed the content and developed an interview guide. Students were asked the following open-ended question: “What is the effectiveness of community-oriented PBL for clinical clerkship?” The interviews were administered by 3 faculty members to 13 students from community-oriented PBL groups during their clinical clerkship. All target students had experienced home visits in their clinical clerkship 1-3 months after community-oriented PBL. The interviewers were trained facilitators from the faculty overseeing community-oriented PBL and conducted thematic analysis. Two researchers (KS and NA) independently read and coded the transcripts. Researcher triangulation was conducted in which the same 2 researchers conducted the analysis and consensus building.

KS and IS, who have extensive experience in qualitative research, defined and regularly discussed the themes and subthemes from the data to ensure the results’ reliability. The cognitive process dimensions to which they corresponded were also evaluated.

### Ethical Considerations

This study was approved by the Ethics Review Committee of the Graduate School of Medicine, Chiba University (approval number 3425). The procedures for obtaining informed consent were explained to the medical students, who were also informed that this study would not affect their grades. All data collected in this study were anonymized to ensure privacy and confidentiality. Participants did not receive any compensation for their participation in this study.

## Results

### Participant Characteristics

**Figure 2:**
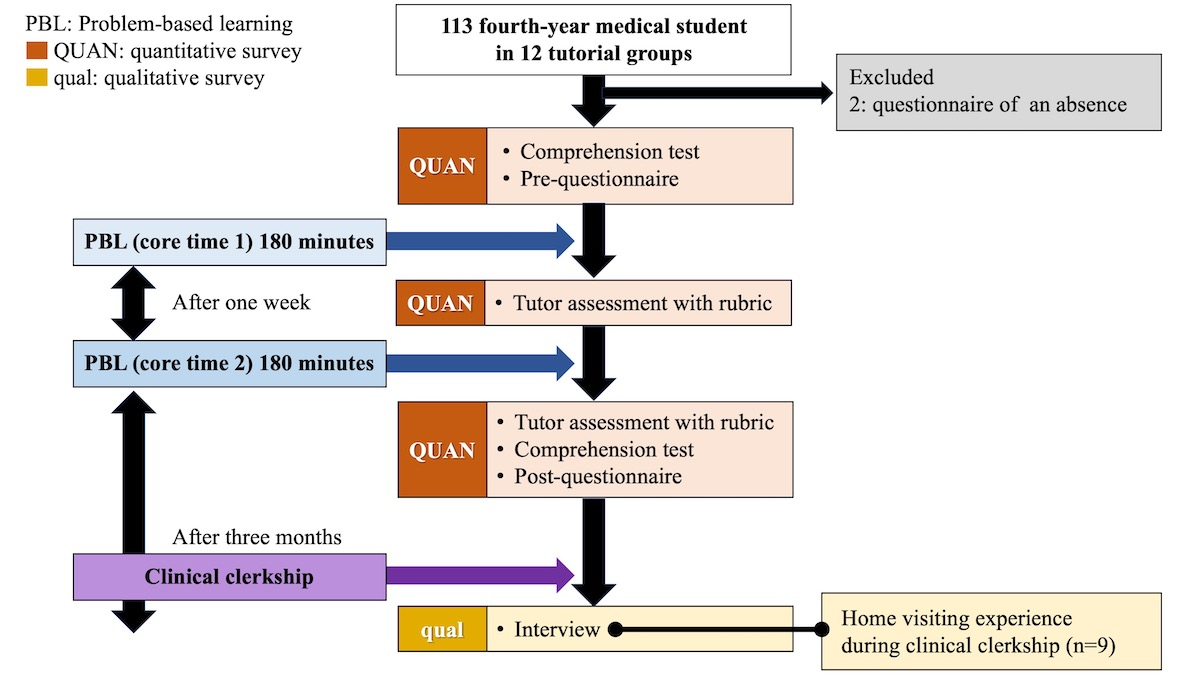
Study flow diagram.

In total, 113 medical students participated in PBL. Of the 113 participants, 71 (62.8%) were male and 42 (37.2%) female. In addition, 2 (1.8%) students were excluded from the survey because they were absent for a core time session; 111 (98.2%) students participated in the quantitative evaluation. The study flowchart is shown in Figure 2.

### Quantitative Measures

#### Comprehension Test

The total comprehension test scores of the students significantly improved after the community-oriented PBL intervention (*P*<.001, effect size *r*=0.528). The total pretest scores had a median of 4.0 (IQR 2.5-5.0), whereas the posttest scores improved to a median of 5 (IQR 4-5). Table 1 provides the pre- and posttest analysis results of each question (Q1-Q5). All questions except Q4 showed significant improvements in the percentage of correct answers.

**Table 1:** Correct answer rates for individual questions in the comprehension test (N=113).

Question number	Pretest correct answers, n (%)	Posttest correct answers, n (%)	*P* value	Effect size (*r*)
1	104 (92.0)	112 (99.1)	.02	2.333
2	69 (61.1)	107 (94.7)	<.001	5.600
3	81 (71.7)	106 (93.8)	<.001	4.640
4	84 (74.3)	93 (82.3)	.19	2.180
5	77 (68.1)	99 (87.6)	<.001	4.310

### Rubric-Based Performance Assessment of Community-Oriented PBL Tutorials

Table 2 presents the results of the rubric-based assessment of the students’ performance in core time 1 and core time 2. The students’ total scores significantly improved from core time 1 (median 38, IQR 33-43) to core time 2 (median 40, IQR 35-44), with *P*<.001 and an effect size (*r*) of 0.516. Regarding individual rubric items, significant improvements were observed in domains such as acquiring knowledge applicable to community health care, developing comprehensive care processes, and fostering self-directed learning. In contrast, no significant improvement was observed in the motivation to learn, whereas interpersonal skills showed a small but statistically significant enhancement.

**Table 2:** Performance assessment of community-oriented PBL^a^ tutorials using a rubric.

Performance items		Core time 1, median (IQR)	Core time 2, median (IQR)	*P* value	Effect size (*r*)
Total score (0-50)		38 (33-43)	40 (35-44)	<.001	0.516
**Domains**					
	1. Acquire knowledge that can be easily recalled and applied in community health care settings (0-10).	8 (7-9)	8 (8-8)	<.001	0.494
	2. Develop an effective, comprehensive community care process (0-10).	8 (6-8)	8 (8-8)	<.001	0.532
	3. Develop self-directed learning methods (0-10).	8 (7-9)	8 (8-8)	<.001	0.553
	4. Motivate myself to learn (0-10).	8 (6-8)	8 (6-8)	.11	0.151
	5. Acquire good interpersonal skills (0-10).	8 (7-9)	8 (7-9)	.03	0.201

^a^PBL: problem-based learning.

### Self-Administered Questionnaire

Results indicated significant changes in students’ perceptions of community health care after participating in community-oriented PBL. For the statement “I am interested in community health care,” the preintervention median score was 3 (IQR 3-4), which increased to 4 (IQR 3-4) post intervention, with a statistical significance of *P*<.001 (*U*=4446.5). Similarly, the median score for the statement “I can envision a community health care setting” improved from 3 (IQR 3-4) preintervention to 4 (IQR 3-4) postintervention, also showing a significant difference, with *P*<.001 (*U*=2589.5).

### Qualitative Measures

#### Follow-Up Interviews During Clinical Clerkships

We explored community-oriented PBL’s impact on medical students’ experiences during home visit consultations. In total, 12 (10.6%) medical students who had not experienced home visits before PBL consented to participate in the interview immediately after acquiring home visit experience during a community clinical clerkship. Through qualitative thematic analysis of the interviews, 7 main themes emerged: “building readiness for home care visit participation,” “understanding and navigating the home care environment,” “professional and personal growth,” “interprofessional collaboration and team dynamics,” “challenges and opportunities in home care,” “community engagement and regional health care systems,” and “ethical considerations and end-of-life care” (Table 3).

**Table 3:** Follow-up interviews and thematic analysis.

Theme	Subthemes
Building readiness for home care visit participation	Broadened understanding of patient care beyond medical intervention Enhanced preparedness for real-world clinical situations Shift in perspective from theoretical knowledge to practical application
Understanding and navigating the home care environment	Insights into the holistic approach required in home visits Observations on the complexities of home care, including resource limitations and patient lifestyles Recognition of the importance of patient and family communication
Professional and personal growth	Aspiration to contribute meaningfully to patient care Development of empathy and emotional intelligence Recognition of the multifaceted role of health care providers in patient support
Interprofessional collaboration and team dynamics	Importance of teamwork and a multidisciplinary approach in patient care Learning from and contributing to the health care team Navigating professional roles and patient relationships
Challenges and opportunities in home care	Adapting PBL^a^ knowledge to address specific patient needs Confronting and managing unique patient care challenges Opportunities for innovative care practices in constrained environments
Community engagement and regional health care system	Enhancing community-oriented medical practice through targeted PBL Gaining insights into community health care needs and resources Understanding the impact of regional characteristics on health care delivery
Ethical considerations and end-of-life care	Deepened understanding of end-of-life care preferences and practices Navigating ethical dilemmas in patient care decisions Valuing patient autonomy and quality of life in care planning

^a^PBL: problem-based learning.

## Discussion

### Principal Findings

This study demonstrated that integrating real-patient videos into community-oriented PBL improves medical students’ knowledge, skills, and attitudes toward community health care. Comprehension test results showed significant improvements in students’ understanding of core concepts, including community-based integrated care systems (Q1), the ICF framework (Q2 and Q3), and holistic, patient-centered care (Q5). These findings highlight students’ enhanced theoretical knowledge essential for community health care practice.

Rubric-based performance assessments revealed notable improvements in 3 key domains:

- Knowledge application (item 1): Students showed improved abilities to recall and apply knowledge in community health care scenarios.
- Developing comprehensive care processes (item 2): Scores reflected stronger skills in designing patient-centered care plans tailored to community settings.
- Self-directed learning (item 3): Students demonstrated enhanced autonomy in planning, monitoring, and reflecting on their learning tasks.

Although interpersonal skills (item 5) improved slightly, no significant changes were observed in the motivation to learn (item 4), indicating areas for potential curriculum enhancement.

Self-assessment questionnaires revealed increased interest in community health care and an improved ability to envision a community health care setting. These results suggest that the intervention can positively influence students’ attitudes and readiness for community health care practice, potentially guiding their career interests toward rural areas.

Qualitative analysis of students’ reflections underscored themes such as readiness for home care visits, professional and personal growth, and community engagement. Students reported a deeper understanding of the complexities of community health care, fostering empathy and patient-centered approaches essential for effective practice in underserved areas.

### Implications of Findings

This study provided valuable insights into how community-oriented PBL, enhanced by real-patient videos, fosters medical students’ ability to conceptualize their future professional roles. The qualitative data indicated that students develop a deeper understanding of the principles and complexities of rural care, including holistic approaches, patient-centered decision-making, and the importance of interprofessional collaboration. These findings suggest that the intervention successfully prepares students to engage with the challenges and rewards of rural and community-based practice.

Importantly, the qualitative analysis revealed that many students began to envision themselves as contributors to rural health care systems. Themes such as “professional and personal growth” and “community engagement” highlighted students’ recognition of their potential roles in underserved areas. This shift was supported by their increased interest in community health care, as measured by the “questionnaire for perceptions of community health care self-assessment.” Postintervention, students reported greater interest and confidence in envisioning community health care settings.

However, although the data indicated a significant attitudinal shift, we lack sufficient evidence to confirm a direct impact on medical students’ long-term career intentions to pursue rural care roles after graduation. Future studies should include longitudinal tracking to assess whether the observed changes in perceptions and interests translate into tangible career decisions. Additionally, research is needed to validate the predictive validity of the self-assessment questionnaire in forecasting students’ career trajectories.

This intervention lays a strong foundation for addressing the global challenge of physician maldistribution by bridging theoretical knowledge with practical applications. However, further investigation is required to understand its long-term influence on medical workforce trends and rural health care outcomes.

### Comparison With the Literature

Our findings will contribute to a growing body of evidence supporting the efficacy of PBL in medical education. Previous studies have demonstrated PBL’s ability to enhance clinical reasoning and decision-making skills [19,21,41]. However, our research added a unique dimension by integrating real-patient videos, which provide authentic learning experiences and contextualize medical knowledge within the framework of community-oriented care. Similar studies have reported that experiential learning approaches, such as case-based learning with audiovisual materials, improve students’ engagement and retention of knowledge [41].

The significant gains in self-directed learning observed in this study echo findings from Matsuyama et al [35], who emphasized the role of contextual attributes in promoting self-regulated learning. Additionally, the qualitative themes identified in our analysis, such as the importance of interprofessional collaboration and navigating the home care environment, align with the existing literature on the competencies required for effective community health care practice [42-48].

### Limitations

Our study has some limitations. First, it was conducted as part of the curriculum of a single medical school, potentially limiting the findings’ applicability to other institutions and geographical settings. Future research should involve multiple institutions to enhance the results’ generalizability. Additionally, the demographic and cultural context of the participants may not fully represent broader populations, especially in countries with different health care challenges and educational frameworks. Second, the absence of a control group makes it difficult to attribute observed improvements solely to the intervention of incorporating real-patient videos into PBL. Our study evaluated a “bundle” of educational strategies, including real-patient videos, the PBL framework, faculty interventions, and testing conditions. We could not measure these components’ differential effects or potential synergistic interactions. Although a randomized controlled trial (RCT) could offer stronger evidence, implementing RCTs in educational settings poses ethical and logistical challenges, such as withholding valuable learning resources from a control group or ensuring equivalent baseline characteristics. To address these limitations, we recommend that future research consider alternative designs to balance rigor and feasibility. Third, the scalability of real-patient videos poses a significant challenge. Producing high-quality real-patient videos requires substantial time, resources, and collaboration among medical educators, health care professionals, and audiovisual specialists. These demands may limit the feasibility of widespread adoption. We suggest collaborative efforts, such as interschool partnerships and the development of shared digital repositories, to distribute production costs and enhance scalability. Fourth, the mixed methods design relied on self-reported measures and reflections, which could introduce a response bias. Future studies could benefit from incorporating objective measures of clinical performance and patient care outcomes. Fifth, the effectiveness of clinical reasoning education via hybrid PBL may vary depending on instructors’ teaching skills. Despite standardized training for tutors, differences in tutor effectiveness may have influenced the consistency of outcomes. We propose further methods to ensure uniformity in future implementations, such as advanced tutor workshops and peer evaluations. Sixth, because the study participants were fourth-year medical students at a single Japanese institution, the results may not be directly generalizable to other populations, such as residents or general physicians, or contexts outside Japan, underscoring the need for further validation. Seventh, the comprehension test used in this study, although developed specifically for the program’s educational objectives, was not formally piloted with a separate cohort. Instead, the test underwent cognitive debriefing with faculty members to ensure clarity, relevance, and alignment with the intended learning objectives. Although this process enhanced content validity, the absence of a formal pilot test may limit the ability to fully validate the test’s reliability and generalizability. Similarly, the 2 items in the self-assessment questionnaire—designed to evaluate medical students’ interest in and ability to envision community health care—were developed based on previous studies and focus group discussions but have not been validated using established scales. This lack of formal validation for the comprehension test and self-assessment questionnaire limits the generalizability and robustness of the findings. Finally, although the qualitative data provided valuable insights into students’ conceptualization of professional roles and their preparedness for community health care settings, the lack of longitudinal data limits our ability to assess the long-term impact of these interventions on career trajectories or practice in rural or underserved areas. Future research should include follow-up assessments to evaluate the sustained influence of such educational interventions on students’ career decisions and professional development.

### Conclusion

Integrating real-patient videos into a community-oriented PBL curriculum shows significant promise in fostering medical students’ interest and competencies in community and rural medicine. Our study demonstrated improvements in knowledge acquisition and application, as indicated by enhanced rubric and comprehension test scores. Moreover, qualitative analysis revealed PBL’s effectiveness in developing essential skills and shaping medical students’ perceptions toward community health care. Although these changes may not directly translate to career decisions, they represent an essential step toward fostering awareness of rural health care needs and aligning medical students’ competencies with the demands of underserved areas. This approach highlights the potential of combining real-patient videos with PBL as an innovative educational strategy to address physician maldistribution and support rural health care systems.

## Appendix

Multimedia Appendix 1 Community-oriented problem-based learning tutor guide.

Multimedia Appendix 2 Information and task sheet.

Multimedia Appendix 3 Comprehension test questions.

Multimedia Appendix 4 Rubric for community-oriented problem-based learning.

Multimedia Appendix 5 Questionnaires for perceptions of community health care self-assessment.

## Abbreviations

ICF: International Classification of Functioning, Disability, and Health
PBL: problem-based learning
RCT: randomized controlled trial

## Data Availability

The datasets generated and analyzed during the study are not publicly available, because they include participants’ personal data, but they are available from the corresponding author upon reasonable request.
