# Supplementary material for: Understanding Community Health Care Through Problem-Based Learning With Real-Patient Videos: Single-Arm Pre-Post Mixed Methods Study
Source: JMIR Med Educ. 2025 Jan 31;11:e68743. doi: 10.2196/68743 (PMC11829178; doi:10.2196/68743)
Supplement: Multimedia Appendix 1 [file mededu_v11i1e68743_app1.docx]

| **Tutor** | **Contents** |
| --- | --- |
| Self-introduction and role assignment  (10 minutes)  Confirmation test  (10 minutes)  Task 1  (20 minutes)  Task 2  (30 minutes)  Task 3  (30 minutes)  Review  (20 minutes)  (End) | - Icebreaking: Encourage group members to designate a moderator and a secretary. - Utilize Google Classroom for the session. - Discussion points should be documented on the Group Task Sheet (Google Docs). The secretary is primarily responsible for documentation, but other members may contribute. - Explain the learning objectives, ground rules, and assessment rubric. - Emphasize that the videos were created with patient cooperation and strictly prohibit the dissemination of this information on social media or any other platform. - Have students explain their answers to the pre-test conducted beforehand, using the Think-Pair method among themselves. Once a consensus is reached among students, the tutor will present the correct answers. - Inform students that the same questions will be posed in Core Time 2. - Present Information Sheet 1 (Clinical Setting) and Video 1 (Clinical Setting, 1 minute 15 seconds) for discussion. - When using Google Maps, inform students that internet access is permitted. - Continue documenting discussion points on the Group Learning Sheet (Google Docs) (and so forth). - Present Information Sheet 2 (Patient Information) for discussion. - Outline the discharge plan (short-term and long-term plans). - Encourage consideration of regional characteristics when creating plans, based on discussion cues #1 (and so forth). - Present Information Sheet 3 (Home Visit Care) and Video 2 (Home Visit Care, 5 minutes 46 seconds) for discussion. - Use the ICF Assessment Worksheet to document ICF assessment and treatment care plans. - Record self-study topics and responsible individuals on the Group Learning Sheet. Information researched should be documented under the "Product" section on the Group Learning Sheet, and presentations will be made in Core Time 2. - Inform students that presentations should not exceed 5 minutes. - Allow for the creation of additional materials but clarify that these will not be evaluated. - Include Rubric Assessment (Individual & Group) on the sheet. |
