# Supplementary material for: Understanding Community Health Care Through Problem-Based Learning With Real-Patient Videos: Single-Arm Pre-Post Mixed Methods Study
Source: JMIR Med Educ. 2025 Jan 31;11:e68743. doi: 10.2196/68743 (PMC11829178; doi:10.2196/68743)
Supplement: Multimedia Appendix 2 [file mededu_v11i1e68743_app2.docx]

**Information and Task Sheet 1 (Core Time 1)**

You are conducting a community clinical clerkship (5th year of medical school) at Hospital A, a core hospital in Katsuura City. Your patient, Mr. O, resides alone on a steep slope along the sea in Katsuura City, Chiba, Japan.

Medical Setting:

Katsuura City, Chiba Prefecture, has a population of approximately 17,000 people, with 40% of residents living in the city being 65 years or older, indicating an aging population.

Hospital A has a total of 290 beds (257 general beds and 33 recuperation beds).

There are approximately 400 staff members, including 17 full-time physicians.

The number of outpatient visits per month attended by full-time physicians is approximately 100, with an additional 10 home visits.

There are seven clinics in Katsuura City, but no other hospitals.

Home visits are conducted, and there are nearby visiting nursing stations and long-term care health facilities.

There are affiliated special nursing homes and low-cost elderly homes within the medical service area.

Hospital B, a nearby comprehensive hospital (with 800 beds and designated as a tertiary emergency medical institution), is 30 minutes by ambulance or 60 minutes by train.

**Task 1:**

Extract from the case sheet and video the difficulties faced by patients/residents living in the community from the perspective of community medicine (e.g., regional characteristics, access to medical care).

The use of Google Maps is permissible for discussing regional characteristics.

The deliberated content should be recorded in the "Group Learning Sheet (Google Document).

**Information and Task Sheet 2 (Core Time 1)**

Patient: Mr. O, 68-year-old male

Family Structure: Living alone

Medical History: Type 2 diabetes, hypertension, dyslipidemia, eczema, obesity (BMI 38)

Present Illness:

Since 2017 Hospitalized at Hospital A for lower leg cellulitis. Subsequent admissions and discharges have occurred intermittently.

October 2022 Hospitalized at Hospital A again for recurrent lower leg cellulitis. Developed acute kidney injury and respiratory distress, transferred to Hospital B.

December 2022 Readmitted to Hospital A for rehabilitation purposes.

Medications: Metformin 500mg tablet, 1 tablet three times daily; Dapagliflozin propylene glycol tablet 10mg, 1 tablet once daily; Amlodipine tablet 10mg, 1 tablet once daily; Losartan tablet 50mg, 1 tablet once daily; Pitavastatin tablet 10mg, 1 tablet once daily; Omeprazole tablet 20mg, 1 tablet once daily; Mecobalamin tablet 500μg, 2 tablets three times daily; Zolpidem tablet 5mg, 1 tablet once daily; Loxoprofen tablet 60mg, 1 tablet as needed for pain.

Lifestyle History: Smoked 20 cigarettes per day until age 62, no alcohol consumption.

**Task 2**

Extract the patient's issues and consider future interventions based on the information provided in the case sheet. Develop a discharge plan (short-term and long-term) accordingly.

Ensure that the discussion incorporates the characteristics of community medicine, shared in Information Sheet 1 and the video, as well as the perspectives of community-based care and patient-centered healthcare.

Record the deliberated content in the "Group Learning Sheet (Google Document).

**Information and Task Sheet 3 (Core Time 1)**

Conducted discharge arrangements with the patient.

Mr. O experiences pain in his lower limbs when walking due to his underlying condition, making walking difficult for him.

His home is approximately 7 kilometers away from the hospital and is situated on a steep slope along the coast. There is no bus service from his home to the hospital, and he does not own a car.

Previously, he relied on friends to drive him to Hospital A for appointments, but due to increased caregiving responsibilities and frequency of visits, this has become difficult.

Due to the difficulty in attending appointments, home visits were initiated.

**Task 3**

Extract the patient's issues and consider future interventions based on the information provided in the case sheet and video. Record these on the ICF (International Classification of Functioning, Disability and Health) evaluation worksheet in the "Group Learning Sheet (Google Document)."

Ensure that the discussion incorporates the characteristics of community medicine, shared in Information Sheet 1 and the video, as well as the perspectives of community-based care and patient-centered healthcare.

**Information and Task Sheet 4 (Core Time 2)**

Two weeks after the last home visit, Mr. O was transported to Hospital A by ambulance.

For two nights ago, he has experienced worsening pain in his right lower leg while walking, and the symptoms have continued to worsen. At the time of consultation, walking is difficult due to pain.

Present symptoms:

Temperature: 37.4°C. Pulse: 88 beats/min, regular. Blood pressure: 162/90 mmHg. Respiratory rate: 14 breaths/min. SpO2: 97% (room air).

No conjunctival pallor or jaundice. Clear breath sounds, no heart murmurs.

Flat abdomen, no surgical scars, normal bowel sounds, soft, no tenderness.

No CVA tenderness.

Indentation edema observed in both lower legs. Redness, warmth, swelling, and tenderness present in the right lower leg. Scaling observed between the second and third toes of both feet.

Examination Findings:

Urinalysis: Protein (+), Glucose (2+), Occult blood (−)

Hematology: RBC 5.2 million, Hb 15.8 g/dL, WBC 11,000/μL, Plt 240,000

Blood chemistry: Fasting blood sugar 182 mg/dL, HbA1c 8.8%, BUN 21.0 mg/dL, Creatinine 1.3 mg/dL, Total bilirubin 1.0 mg/dL, AST 52 IU/L, ALT 82 IU/L, γ-GTP 56 IU/L, CRP 9.2 mg/dL.

Mr. O was urgently hospitalized on the same day.

**Task 4**

Extract the patient's issues, particularly from a psychosocial perspective, based on the information provided in the case sheet and video, and formulate an inpatient treatment plan.

Ensure that the discussion incorporates the characteristics of community medicine, shared in Information Sheet 1 and the video, as well as the perspectives of community-based care and patient-centered healthcare.

Record the considerations on the "Group Learning Sheet (Google Document).

**Information and Task Sheet 5 (Core Time 2)**

Post-admission Progress:

Two sets of blood cultures were taken, and antibiotic therapy with cefazolin 1g/day was initiated. Subsequently, the redness, swelling, warmth, and pain in the lower leg improved. All blood cultures were negative.

Diet therapy was implemented with 1600 kcal/day and 6 g/day of salt intake. Additionally, nutritional guidance was provided by a dietitian.

Daily walking exercises and massages were conducted, resulting in a weight loss of 7 kg due to dietary management.

**Task 5**

Extract the patient's issues and consider future interventions based on the information provided in the case sheet. Document these on the ICF (International Classification of Functioning, Disability and Health) Evaluation Worksheet in the "Group Learning Sheet (Google Document)."

The ICF Evaluation Worksheet should be refined based on the considerations made thus far, building upon what was created in Core Time 1.

Ensure that the discussion incorporates the characteristics of community medicine, shared in Information Sheet 1 and the video, as well as the perspectives of community-based care and patient-centered healthcare.
