# Supplementary material for: Understanding Community Health Care Through Problem-Based Learning With Real-Patient Videos: Single-Arm Pre-Post Mixed Methods Study
Source: JMIR Med Educ. 2025 Jan 31;11:e68743. doi: 10.2196/68743 (PMC11829178; doi:10.2196/68743)
Supplement: Multimedia Appendix 3 [file mededu_v11i1e68743_app3.docx]

**Question 1. Select the correct statement about the community-based integrated care system.**
a. Applications for long-term care certification are submitted through medical institutions.

b. Municipal staff determine the care level solely through assessment surveys.
c. Problem-solving capabilities include self-help, mutual aid, community, public, and private aid.
d. Care managers are responsible for preparing the primary physician's opinion form.
e. The community-based integrated care system refers to a community's comprehensive support and service delivery framework.

**Question 2. Select the correct statement about the International Classification of Functioning, Disability, and Health (ICF).**
a. The components of participation include only “participation restrictions.”
b. The activity components include “activity” and “activity limitations.”
c. The components of body functions and structures include “mental functions” and “functional structures.”
d. Personal factors refer to the physical environment in which people live.
e. Examples of environmental factors include lifestyle, social background, and past and present experiences.

**Question 3. Select the incorrect combination of components and examples in ICF evaluation.**
a. Body functions and structures – The patient has reduced cardiac function.
b. Participation – The patient was a homemaker managing household chores and caregiving for her husband.
c. Activity – The patient requires assistance with grooming, toileting, and bathing.
d. Health condition – The patient experienced a myocardial infarction and received treatment but faces a risk of recurrence.
e. Personal factors – The patient’s daughter works late into the night, making her unavailable for household responsibilities.

**Question 4. Select the correct statement about health, disease, and illness perspectives in patient-centered care.**
a. Illness refers to meaning, purpose, and a sense of fulfillment.
b. Health perspectives refer to medical history, physical examinations, and diagnostic tests.
c. Understanding illness involves assessing emotions, expectations, interpretations, and impacts.
d. Understanding health perspectives involves identifying desired tests or explanations from the patient.
e. Discussing illness enhances patients’ knowledge and promotes prevention and health.

**Question 5. Select the correct combination for a holistic understanding of patient-centered care.**
a. Individual – Family
b. Proximal context – Development
c. Proximal context – Life cycle
d. Distal context – Social support
