# Supplementary material for: Understanding Community Health Care Through Problem-Based Learning With Real-Patient Videos: Single-Arm Pre-Post Mixed Methods Study
Source: JMIR Med Educ. 2025 Jan 31;11:e68743. doi: 10.2196/68743 (PMC11829178; doi:10.2196/68743)
Supplement: Multimedia Appendix 4 [file mededu_v11i1e68743_app4.docx]

1. **Acquire knowledge that can be easily recalled and applied in community healthcare settings**

| **0 Points** | **2 Points** | **4 Points** | **6 Points** | **8 Points** | **10 Points** |
| --- | --- | --- | --- | --- | --- |
| No knowledge. | Only very basic knowledge. | Has knowledge but cannot recall it timely. | Occasionally can apply knowledge. | Often can apply knowledge to discussions. | Can fully apply knowledge to discussions. |

1. **Develop an effective comprehensive community care process**

| **0 Points** | **2 Points** | **4 Points** | **6 Points** | **8 Points** | **10 Points** |
| --- | --- | --- | --- | --- | --- |
| Does not understand the process of community care. | Often makes statements ignoring the process of community care. | Statements do not contribute to the promotion of community care. | Occasionally makes statements promoting community care. | Often makes suggestions promoting community care with understanding of the process. | Always makes statements promoting community care with a good understanding of the process. |

1. **Develop self-directed learning methods**

| **0 Points** | **2 Points** | **4 Points** | **6 Points** | **8 Points** | **10 Points** |
| --- | --- | --- | --- | --- | --- |
| No preparation or study of tasks. | Preparation and study of tasks are very inadequate. | Has prepared and studied but not sufficiently. | Prepared and studied and can participate in discussions. | Preparation and study are helpful for discussions. | Preparation and study are very helpful for discussions. |

**4. Motivate myself to learn**

| **0 Points** | **2 Points** | **4 Points** | **6 Points** | **8 Points** | **10 Points** |
| --- | --- | --- | --- | --- | --- |
| No motivation for learning, hinders discussion. | Lacks motivation for learning but does not hinder discussion. | Has motivation for learning but speaks infrequently. | Motivated for learning and participates in discussions. | Highly motivated for learning and takes on roles such as moderator. | Highly motivated for learning and enhances the learning motivation of the entire group. |

**5. Acquire good interpersonal skills**

| **0 Points** | **2 Points** | **4 Points** | **6 Points** | **8 Points** | **10 Points** |
| --- | --- | --- | --- | --- | --- |
| No dialogue with group members. | Can express own opinions but intentions are not conveyed. | Can express own opinions but sometimes lacks clarity. | Pleasantly expresses own opinions and makes members understand. | Can sufficiently make members understand own opinions. | Excellent communication skills, able to effectively consolidate conflicting opinions. |
