# Supplementary material for: Understanding Community Health Care Through Problem-Based Learning With Real-Patient Videos: Single-Arm Pre-Post Mixed Methods Study
Source: JMIR Med Educ. 2025 Jan 31;11:e68743. doi: 10.2196/68743 (PMC11829178; doi:10.2196/68743)
Supplement: Multimedia Appendix 5 [file mededu_v11i1e68743_app5.docx]

1. I am interested in community healthcare
2. strongly disagree
3. disagree
4. neutral
5. agree
6. strongly agree
7. I can envision a community healthcare setting
8. strongly disagree
9. disagree
10. neutral
11. agree
12. strongly agree
